# miRNA Expression Profile in Primary Limbal Epithelial Cells of Aniridia Patients

**DOI:** 10.1167/iovs.66.1.20

**Published:** 2025-01-09

**Authors:** Mahsa Nastaranpour, Shweta Suiwal, Tanja Stachon, Fabian N. Fries, Maryam Amini, Berthold Seitz, Eckart Meese, Nicole Ludwig, Nóra Szentmáry

**Affiliations:** 1 Dr. Rolf M. Schwiete Center for Limbal Stem Cell and Congenital Aniridia Research, Homburg/Saar, Germany, Saarland University, Homburg/Saar, Germany; 2 Department of Ophthalmology, Saarland University Medical Center, Saarland University, Homburg/Saar, Germany; 3 Department of Human Genetics, Saarland University, Homburg/Saar, Germany; Center for Human and Molecular Biology, Saarland University, Homburg/Saar, Germany

**Keywords:** aniridia, microRNA (miRNA), primary limbal epithelial cells (pLECs)

## Abstract

**Purpose:**

This study evaluates the microRNA (miRNA) expression profile in primary limbal epithelial cells (pLECs) of patients with aniridia.

**Methods:**

Primary human LECs were sampled and isolated from 10 patients with aniridia and 10 healthy donors. The miRNA profile was analyzed using miRNA microarrays. The biological roles of miRNA-validated target genes were delineated in silico by the enrichment analyses of the gene ontology (GO) and Kyoto Encyclopedia of Genes and Genomes (KEGG) pathway. The expression of the most deregulated miRNAs was analyzed using quantitative real-time PCR (qRT-PCR).

**Results:**

Microarray analysis revealed 10 differentially expressed miRNAs in pLECs of patients with aniridia relative to healthy controls (fold change = ≤ –2 or ≥ +2), nevertheless these were only differentially expressed using an unadjusted *P* value < 0.05. The qRT-PCR validation confirmed the significantly altered expression of miR-138-5p in pLECs of patients with aniridia (*P* = 0.005). In silico GO analysis of miR-138-5p target genes revealed the potential biological functions of miR-138-5p in regulating various cellular and molecular processes, including the positive regulation of cell motility, G1/S phase cell cycle transition, and cell migration, as well as the negative role in regulating epithelial cell differentiation. Pathway analysis highlighted the main involvement of the PI3K-Akt, Hippo, Wnt, Focal adhesion, cAMP, p53, IL-17, Jak-STAT, and MAPK-signaling pathways.

**Conclusions:**

This study revealed miRNA expression profile in pLECs of patients with aniridia using miRNA microarray and identified miRNAs that had not been previously reported for aniridia LECs. Our study also provides functional and pathway information that can be used to predict possible mechanism of miRNA function in LECs, thereby bridging the gap in the pathogenesis of AAK studies.

Aniridia is a rare, congenital, panocular disease, mainly characterized by a variable degree of iris hypoplasia or the total absence of obvious iris tissue.^1^^,^^2^ In most aniridia cases, there is a mutation or deletion in the paired box 6 gene (*PAX6*), therefore, one copy of the *PAX6* gene loses its function (haploinsufficiency).^3^^–^^6^ The estimated incidence of congenital aniridia ranges from 1 in 64,000 to 1 in 100,000 and can occur either as an isolated condition or in association with other syndromes.^7^^–^^9^

Aniridia associated keratopathy (AAK) is the term given to progressive opacification of the cornea in congenital aniridia. In AAK, there is progressive loss of limbal epithelial stem cell (LESC) function, nonhealing epithelial defects, corneal neovascularization, inflammation, and ingrowth of conjunctival tissue toward the corneal center. AAK alone is also responsible for progressive vision loss in affected patients with aniridia, impacting 80% of patients with varying degrees of severity.^10^^–^^16^

MicroRNAs (miRNAs) are a class of small non-coding RNAs (approximately 20–24 nucleotides), which regulate most human coding genes at the posttranscriptional level through a specific binding to their target mRNA. Numerous studies have indicated that miRNAs have a role in controlling corneal neovascularization.^17^^–^^23^ Our previous study demonstrated dysregulation of miRNAs in conjunctival cells of patients with aniridia with different AAK severity grades, with miR-204-5p and miR-205-5p being downregulated in conjunctival cells of these patients.^24^ The miR-204-5p has been described to regulate angiogenesis in corneal epithelial cells through regulation of angiopoietin-1 (Angpt1).^25^^–^^27^ Abbasi et al. reported that short-term miR-204-5p treatment effectively suppresses VEGFA and ANGPT1 and enhances PAX6 expression in multiple corneal epithelia.^27^ Another study described that inhibition of miR-205-5p resulted in impaired wound healing of corneal epithelial cells.^28^

In the pancreatic cells of the aniridia mouse model (*Pax6^SeyDey^*^−/+^ mouse), miR-7 regulates the PAX6 protein level through complementary binding to the 3′ untranslated region (3′UTR) of PAX6.^29^^,^^30^ Other studies have reported that PAX6 is necessary for regulating miR-204 expression during lens development.^31^ These findings suggest that PAX6 may either regulate specific miRNAs or, conversely, be regulated by several miRNAs.

Given the crucial role of miRNAs in protein regulation in AAK and the current lack of knowledge regarding miRNA expression in limbal cells, this study aimed to determine the miRNA expression profile in primary limbal epithelial cells (pLECs) of patients with aniridia and to identify miRNAs potentially associated with AAK pathogenesis.

## Materials and Methods

### Ethical Considerations

Our study was approved by the Ethics Committee of Saarland/Germany (No 21/21) and followed regulations of the Declaration of Helsinki. Informed consent was obtained from all participants.

### Study Subjects

Ten small limbal pieces of 10 patients with aniridia (age = 34.9 ± 10.27 years, range = 17–46 years, 6 (60%) male patients, 5 limbal biopsies (age = 64.25 ± 2.75 years, range = 61–67 years, and 3, 80% male patients), and 5 corneoscleral donor rims as a control group (age = 56.6 ± 0.4 years, range = 56–57 years, three, 60% male patients, from the Klaus Faber Center for Corneal Diseases including Lions Eye Bank) have been used for miRNA profiling. The clinical and genetic data of patients with AAK are listed in Table 1.

**Table 1.** Demographics and Genetical Information of the Analyzed Congenital Aniridia Subjects

Subject Number	Gender	Age	Mutation Type	Functional Consequence (Predicted)	Affected Region	DNA Change	Protein Change	AAK Grade
1	F	27	PTC	NMD inducing	Exon 10	c.781C>T	p. (Arg261*)	5
2	F	51	PTC	NMD inducing	Exon 5	c.33delC	p.Glyl2Valfs*19	4
3	M	41	Splice site		Intron 5	c.1423C>G		3
4	M	24	n/a	Frameshift	Exon10	c.798_819dup	p.(Gln274LysfsTer17)	1
5	F	16	Deletion	n/a	Exon 11-15+ELP4 Exon 9	PAX6	n/a	4
6	M	37	Nonsense		Exon 11	c.949C>T	p.(Arg317*)	3
7	F	25	Nonsense		Exon 4	c.4C>T	p.(Gln2*)	3
8	M	19	Nonsense		Exon 5	c.120C>A	p.(Cys40*)	4
9	M	43	Splice site	Removing the stop codon	Intron 12	(c.1226-2A>G)	n/a	4
10	M	36	Nonsense-mutation, PTC	Truncated protein	Exon 10	c.829C>T	p.(Gln277*)	3

Aniridia associated keratopathy (AAK) was graded according to Lagali et al.^11^ In the sample set for patient numbers 1 through 5, the microarray has been performed and the microarray data was first validated using RT-qPCR. In the patients numbers 6 through 10, a repeat validation was performed, followed by the validation of the target genes.

n/a, not available; NMD, nonsense-mediated RNA decay; PTC, premature termination codon.

### Aniridia and Healthy Primary Limbal Epithelial Cell Cultures

The pLECs were isolated and cultured, as described previously by Latta et al.^32^ In brief, pLECs were obtained from small 1 × 2 mm limbal biopsies surgically removed from patients with aniridia or from the corneoscleral rims of healthy donors. First, the limbal tissue pieces were punched out of the limbal region of corneoscleral rings using a 1.5 mm punch (BP-15F KAI Europe GmbH, Solingen, Germany). The limbal pieces of both patients with aniridia and healthy donors were incubated overnight at 37°C with 500 µL collagenase A (4 mg/mL; Roche Pharma AG, Basel, Switzerland). After 24 hours of digestion, pLECs were filtered through 40 µm Flowmi cell strainers (Cat. No.: H13680-0040, Bel-Art Products, Wayne, NJ, USA) to remove fibroblasts, washed, and the cell clusters were dissolved using 2.5 mL trypsin-EDTA solution (Cat. No.: T3924, Sigma-Aldrich GmbH, Deisenheim, Germany). The isolated cells were seeded into a single well of a 24-well plate and were cultured in keratinocyte serum-free medium (Cat. No.: 17005-034, KSFM; Gibco, Carlsbad, CA, USA), supplemented with 50 µg/mL bovine pituitary extract (BPE; Cat. No.: 13028-014; Gibco Life Technologies, Paisley, UK), 5 µg/µL epidermal growth factor (EGF; Cat. No.:10450-013; Gibco Life Technologies, Paisley, UK), and 100 U/mL penicillin/streptomycin (Cat. No.: P4333; Sigma Aldrich GmbH, Deisenheim, Germany). Before reaching confluence, the culture medium was exchanged every 3 days. Thereafter, at 90% confluency, we passaged the cells into one well of a 6-well-plate, using 500 µL trypsin-EDTA-solution and cultured those in 3 mL of the above supplemented KSFM. Then, the cells were passaged 2 times and were either used directly for microarray measurements or were cryopreserved in Cryo-SFM- medium (Gibco, Carlsbad, CA, USA) and stored at –80°C until further use.

### RNA Isolation and Quality Control

RNA was extracted using RNA/DNA/Protein Purification Plus Micro Kit (Cat. No.: 47700; Norgen Biotek, Thorold, Ontario, Canada), according to the manufacturer's recommendations, including the optional step of on column DNA digestion to generate higher quality RNA. In brief, the cells were homogenized and lysed by adding 300 µL lysis buffer (SKP-buffer of RNA/DNA/Protein Purification Plus Kit; Norgen Biotek, Thorold, Ontario, Canada) for 15 minutes at room temperature. Afterward, the cell lysates were loaded onto a gDNA purification column and were centrifuged at 5200 × g for 2 minutes. After the centrifugation step, the flowthrough containing RNAs and the proteins were retained. Thereafter, RNA was precipitated by adding 60 µL 96% to 100% ethanol to every 100 µL flowthrough and loaded onto the RNA/Protein Purification Column. The column was washed thrice with wash solution and, subsequently, the total RNAs including miRNAs were eluted in 30 µL RNA Elution Buffer by centrifugation at 200 g for 2 minutes. For quality control, RNA concentration was measured using Nanodrop 2000 spectrophotometer (Thermo Fisher Scientific, Waltham, MA, USA), and RNA integrity was assessed by determining the corresponding RNA integrity number (RIN) values using the RNA 6000 Nano kit on an Agilent 2100 Bioanalyzer instrument (Agilent Technologies, Santa Clara, CA, USA).

### miRNA Microarray Profiling of Aniridia and Healthy Primary Limbal Epithelial Cells

The miRNA expression profile in pLECs of 5 patients with aniridia and 5 healthy controls was monitored using the SurePrint G3 8 × 60k miRNA microarray (miRBase release 21.0; Agilent Technologies, Santa Clara, CA, USA), which enables detection of 2549 human mature miRNAs and contains miRNA Complete Labeling and Hyb Kit (Agilent Technologies, Santa Clara, CA, USA). Briefly, 100 ng of RNA was dephosphorylated, labeled with Cy3-pCp, and then hybridized to the array at 55°C, 20 rpm for 20 hours. After 2 washes, the slides were scanned using an Agilent Microarray Scanner (G2505C; Agilent Technologies, Santa Clara, CA, USA) with 3-µm resolution in double-pass mode. The raw expression data were extracted using the manufacturers’ Feature Extraction Software (Agilent Technologies, Santa Clara, CA, USA; version 10.10.1.1) and were checked for the quality and distribution of miRNAs.

#### Statistical Analysis of Microarray Data

Analysis of microarray data has been performed with GeneSpring software (version 14.9.1; Agilent Technologies, Santa Clara, CA, USA). Prior to statistical analysis, extracted raw data were log_2_ transformed and normalized using quantile normalization. Thereafter, miRNAs were filtered for expression using the “detected” flag and only miRNAs were retained that showed expression in at least 50% of the samples. To identify the significant differentially expressed miRNAs, we used unpaired *t*-test with Benjamini-Hochberg false discovery rate (FDR) adjustment to correct *P* values for multiple testing. Only differentially expressed miRNAs were considered, which demonstrated at least a 2-fold decreased or increased mean expression value and an adjusted *P* value < 0.05 in the *t*-test.

#### Target Genes of Dysregulated miRNAs

In order to find functional categories, biological processes, or ontology terms enriched by target genes of the deregulated miRNAs, the online tool miRTargetLink (https://ccb-compute.cs.uni-saarland.de/mirtargetlink2) was used in conjunction with strong validated, that is, with reporter assay, Western blot, and/or quantitative PCR (qPCR) validated, targets based on miRTarbase (https://mirtarbase.cuhk.edu.cn/∼miRTarBase/miRTarBase_2022/php/index.php).^33^^,^^34^

#### Pathway Analysis

The next step after specifying the validated miRNA target genes was to identify the target gene pathways. Therefore, we used the GeneTrail (version 3.2) analysis platform,^35^ a web-based application that allows specifying enriched functional categories and pathways in gene sets. We focused our analysis on annotated pathways from the KEGG database. In this tool, the validated miRTarget genes are summarized in various biological categories and are statistically evaluated whether a set of genes shows a significant enrichment in the considered categories.

#### Gene Ontology Classification Analysis

To uncover the function of the validated gene targets of the significantly upregulated miRNA and to further test the biological links in co-regulated genes, we applied Gene Ontology (GO) classification, including biological process (GO-BP), molecular function (GO-MF), and cellular component (GO-CC) analysis. The genes identified by miRTarbase and miRTargetLink online tools were submitted to GeneTrail (version 3.2), using the local human KEGG database.

### Reverse Transcription Quantitative Real-Time PCR Validation of miRNAs and miRNA Target Gene Expression in Aniridia Primary Limbal Epithelial Cells and Healthy Controls

To validate the array data, we performed reverse transcription-quantitative PCR (RT-qPCR) on the deregulated miRNAs. For this analysis, we utilized the original sample set from the previous microarray study, supplemented with five additional samples. These miRNAs were selected based on their changed expression level in aniridia pLECs (miR-409-3p, miR-495-3p, miR-127-3p, miR-493-5p, miR-382-5p, miR-634, miR-193a-5p, and miR-138-5p). Due to the unavailability of RNA from the original sample set, the investigation of miR-138-5p target genes was conducted solely using the additional sample set. The cDNA synthesis was performed by miRCURY LNA miRNA RT Kit (Cat. No.: 339340; Qiagen, Hilden, Germany), using PCR Thermocycler CFX Connect (Bio-Rad Laboratories, Munich, Germany), based on the provided protocol by the manufacturer. The qPCR was performed in triplicates using miRCURY LNA miRNA SYBR Green PCR kit (Cat. No.: 339345; Qiagen GmbH, Hilden, Germany) using QuantStudio 5, following the manufacturer’s protocol. The level of gene expression was determined using QuantStudio design and analysis software with miR-103a-3p and snRNA RNU6 (Qiagen, Hilden, Germany) as endogenous controls. Relative expression (fold change) was calculated with the ΔΔCt method (Reference: PMID: 11846609). Primers used for miRNAs validation are displayed in Supplementary Table S1. Primer pairs used for miR-138-5p target gene investigation are displayed in Supplementary Table S2.

### Statistical Analysis of miRNA Expression Data

The miRNA expression values were exported, and fold changes were calculated in Excel (Microsoft, Redmond, WA, USA). The normal distribution of fold change values for each miRNA was assessed using the Shapiro-Wilk test. For normally distributed values, *P* values were calculated using an unpaired Student’s *t*-test. For non-normally distributed values, the nonparametric Mann-Whitney test was applied. Nonparametric Kruskal-Wallis test (ANOVA), followed by post hoc analysis Dunn's test for multiple comparisons, was used to analyze subgroups of AAK grades in comparison with controls. A *P* value of less than 0.05 was considered statistically significant. Data analysis and graph preparation were performed using GraphPad Prism 7.04 software (San Diego, CA, USA).

## Results

### miRNA Expression Profile of Aniridia and Healthy Primary Limbal Epithelial Cells

We evaluated miRNA signatures from pLECs of patients with aniridia. We measured the expression of 2459 miRNAs using microarray technology, based on miRBase version 21. Out of all 2459 tested miRNAs, 482 were expressed in more than 50% of samples above the background level. Selecting these 482 miRNAs, our approach enabled us to exclude miRNAs expressed only at background levels. The rationale behind this method was to concentrate on the 482 miRNAs expressed above background level, out of the 2459 miRNAs tested, and present in at least 50% of the samples. This method was described previously.^24^ Then, we performed an unpaired *t*-test to identify miRNAs that showed a differential expression in the aniridia samples compared with the controls. There were no significantly deregulated miRNAs after FDR adjustment for multiple testing, probably due to low sample sizes. However, we observed a difference in the expression of some miRNAs before correction of the *P* values (unadjusted *P* value < 0.05).

**Figure 1.**
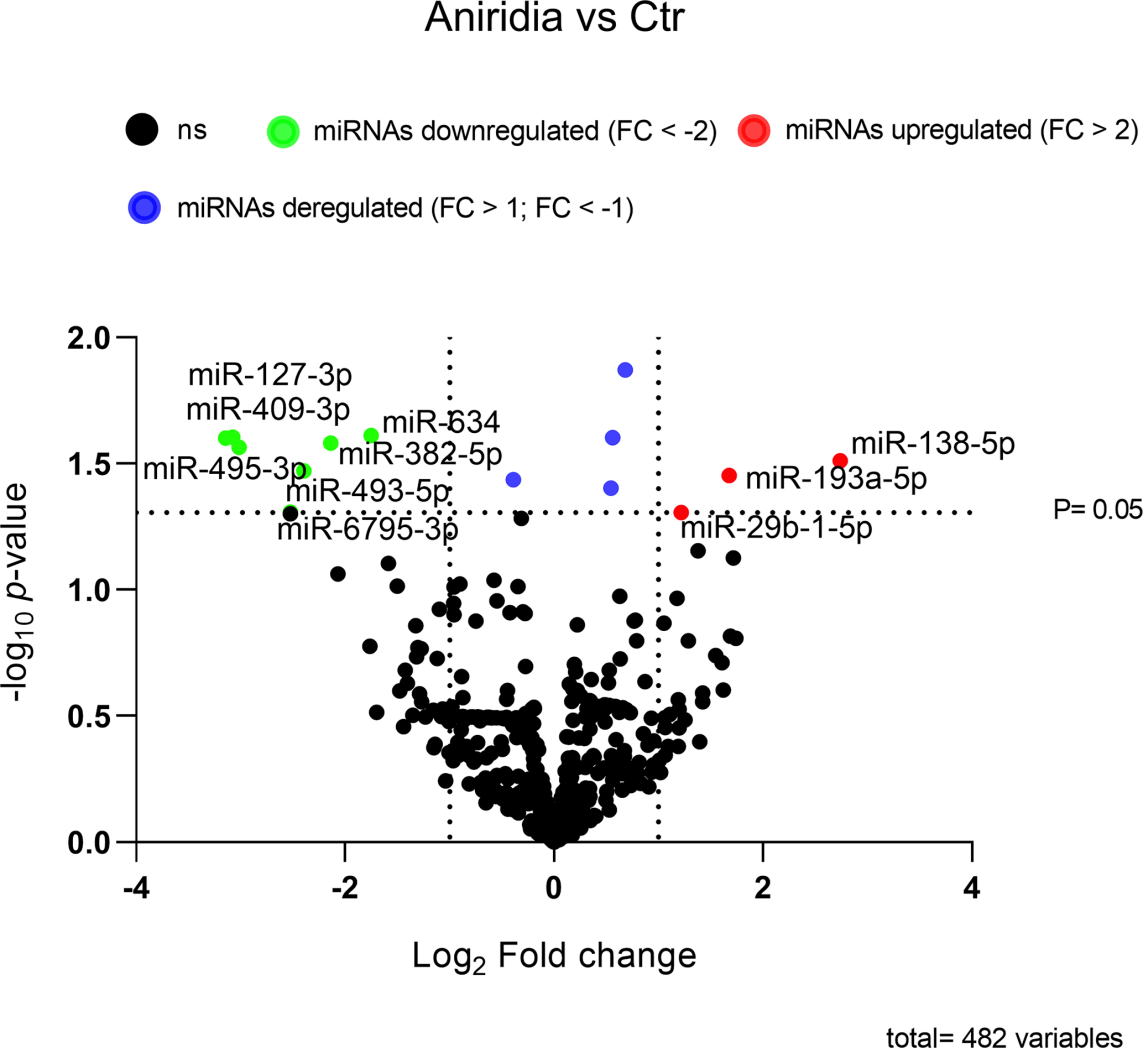
**Volcano plot for the differentially expressed miRNAs in primary limbal epithelial cells (pLECs) of aniridia subjects versus controls.** The Volcano plots indicate on the y-axis the negative log_10_ raw *P* value of the *t*-test and on the X-axis the log_2_ fold change. Significant miRNAs are indicated in *red* (overexpressed in pLECs) or *green* (reduced expression in pLECs), and *blue* (deregulated miRNAs with fold change = > 1 and fold change = < –1). The *horizontal black dotted line* separates miRNAs that are significant (above the line; unadjusted *P* < 0.05) and the miRNAs that are not significant anymore (below the *black dotted line*).

**Figure 2.**
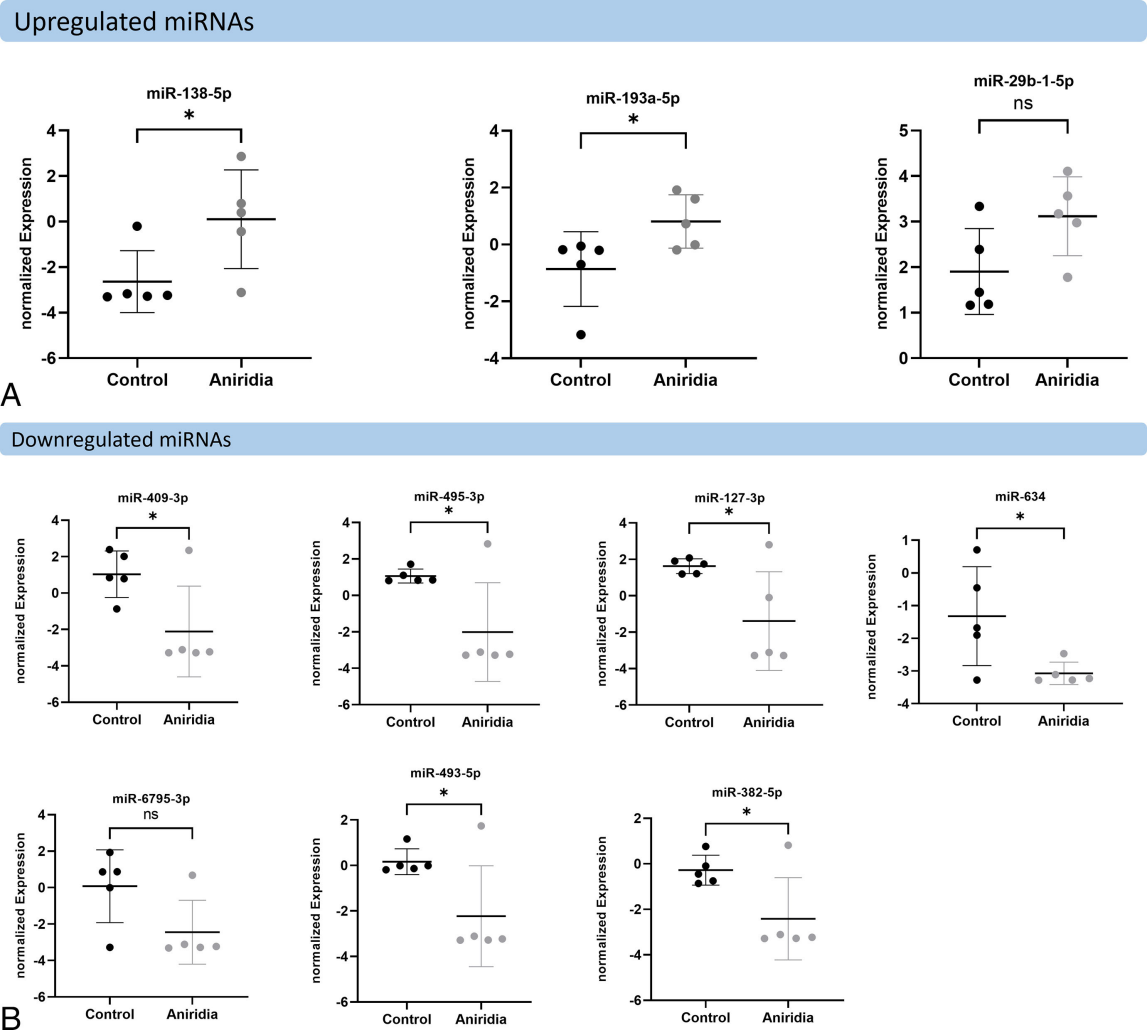
**Normalized miRNA expression of selected significantly upregulated miRNAs** (**A**) **and downregulated miRNAs** (**B**) (**see** **Table 2**) **in aniridia primary limbal epithelial cells (pLECs) compared with healthy controls, with unadjusted *P* values.** Expression values are represented as scatter plot along with mean and SD. Statistical analysis has been performed using two-tailed *t*-test. n.s., not significant; * unadjusted *P* < 0.05.

In total, we identified 10 miRNAs of a total of 482 miRNAs, that were significantly deregulated in aniridia samples compared with controls (unadjusted *P* < 0.05; Fig. 1). Among these 10 significant miRNAs, 3 miRNAs were upregulated, and 7 miRNAs were downregulated in aniridia with 4 miRNAs showing more than 5-fold expression difference (Fig. 2). The most strongly downregulated miRNAs were miR-409-3p, miR-495-3p, and miR-127-3p with an 8-fold reduction of their expression in aniridia samples, whereas the most strongly upregulated miRNA was miR-138-5p with a 6-fold increased expression compared with controls, as detailed in Table 2.

**Table 2.** The Down- and Upregulated miRNAs in Congenital Aniridia Primary Limbal Epithelial Cells (pLECs)

Upregulated miRNAs in Aniridia pLEC				Downregulated miRNAs in Aniridia pLEC			
	Fold Change	Raw *P* Value	Corrected *P* Value		Fold Change	Raw *P* Value	Corrected *P* Value
miR-138-5p	2.32	0.04	1	miR-634	−3.36	0.02	1
miR-193a-5p	3.19	0.03	1	miR-382-5p	−4.40	0.02	1
miR-29b-1-5p	6.67	0.03	1	miR-493-5p	−5.26	0.03	1
				miR-6795-3p	−5.75	0.04	1
				miR-127-3p	−8.09	0.02	1
				miR-495-3p	−8.42	0.02	1
				miR-409-3p	−8.83	0.02	1

Samples were ordered by increasing fold changes.

### Validation of Selected miRNAs by RT-qPCR in Aniridia and Healthy Primary Limbal Epithelial Cells

First, the microarray data were validated using RT-qPCR in the same sample set that was used for array analysis. In detail, we chose strongly downregulated miR-495-3p, miR-409-3p, miR-127-3p, miR-493-5p, miR-634, and miR-382-5p, and strongly upregulated miR-138-5p and miR-193a-5p for the validation. In patients with aniridia versus healthy controls, miR-409-3p, miR-495-3p, and miR-127-3p showed a downregulation with 8.83-, 8.42-, and 8.09-fold change in the array analysis and downregulation with 0.68-, 0.86-, and 0.68-fold change in the RT-qPCR analysis, respectively. Likewise, a downregulation was found for the miR-493-5p and miR-382-5p with 5.26- and 4.40-fold changes, respectively, in the array analysis, and 0.56- and 0.63-fold downregulation, respectively, in the RT-qPCR analysis. The miR-634 was downregulated in microarray with 3.36-fold change, however, it showed upregulation with 1.68-fold change in RT-qPCR analysis. The discrepancy in miR-634 regulation observed between the microarray and RT-qPCR results may stem from the low expression levels of miR-634 in limbal epithelial cells. In RT-qPCR, the mean Ct value for miR-634 was above 35, resulting in high variability among samples and potentially impacting the accuracy of fold change analysis.

**Figure 3.**
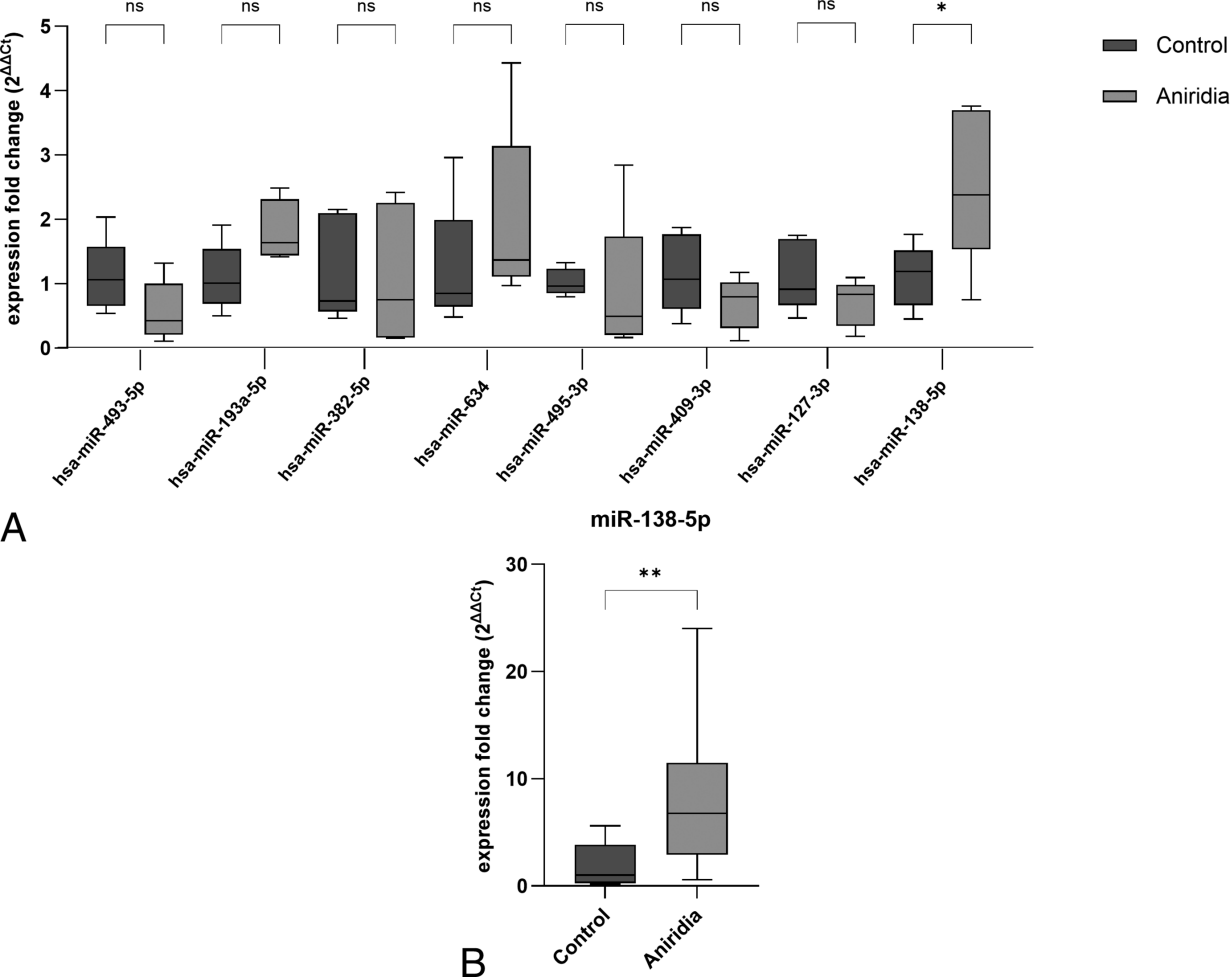
**RT-qPCR validation of deregulated miRNAs.** (**A**) The box-whisker plot shows on the y-axis the expression fold change of deregulated miRNAs in primary limbal epithelial cells (pLECs) of patients with aniridia compared with healthy controls, using RT-qPCR. Apart from miR-138-3p, there was no significant difference in expression of other miRNAs in the RT-qPCR results using unpaired two-tailed *t*-test (*n* = 5). (**B)** RT-qPCR analysis of both the primarily used and the additional aniridia sample set revealed a significant difference in miR-138-5p expression between aniridia and control samples. The box plots represent the second and third quartile, the whiskers indicate the minimum and maximum values. n.s., not significant, * *P* < 0.05, ** *P* < 0.01, *n* = 10.

The upregulation of miR-138-5p in the aniridia samples versus controls was found in both in the array and the RT-qPCR analysis, with 6.67- and 2.55-fold changes, respectively (Table 3). Therefore, the direction of deregulation was generally confirmed for all tested miRNAs between patients with aniridia and healthy controls except miR-634, nevertheless, only miR-138-5p upregulation in RT-qPCR results were statistically significant (*P* = 0.03; Fig. 3a).

**Table 3.** Reverse Transcription Quantitative Real-Time PCR (RT-qPCR) Validation of miRNA Expression, in Aniridia Primary Limbal Epithelial Cells (pLECs)

	Fold Change		Direction of Regulation		*P* Value	
miRNA	Array	RT-qPCR	Array	RT-qPCR	Array	RT-qPCR
miR-495-3p	−8.42	0.86	Down	Down	0.02	0.76
miR-409-3p	−8.83	0.68	Down	Down	0.02	0.18
miR-127-3p	−8.09	0.68	Down	Down	0.02	0.18
miR-493-5p	−5.26	0.56	Down	Down	0.03	0.14
miR-382-5p	−4.40	0.63	Down	Down	0.02	0.87
miR-634	−3.36	1.68	Down	Up	0.02	0.35
miR-138-5p	6.67	2.55	Up	Up	0.03	**0.005**
miR-193a-5p	3.19	2.54	Up	Up	0.04	0.07

Subsequently, miR-138-5p validation was conducted again using RT-qPCR on an additional congenital aniridia sample set to assess the generality of miR-138-5p dysregulation. The statistically significant upregulation of miR-138-5p in the new aniridia samples compared with controls was reconfirmed by RT-qPCR (Fig. 3b). Consequently, miR-138-5p was selected for further investigation to identify potential target genes and perform GO and pathway enrichment analyses.

Furthermore, we analyzed the expression of miR-138-5p across different AAK grade subgroups (AAK grades 1–3 and AAK grades 4–5) in all the aniridia samples used in the study. However, no significant differences in miR-138-5p expression were observed between the AAK grade subgroups (*P* ≥ 0.07).

### Target Genes of miR-138-5p

To gain more insight in the function of the upregulated miR-138-5p in association with AAK we identified potentially relevant miRNA-mRNA target interactions by using miRTargetLink2, which is using the miRTarBase as the data resource, which has been proven to be the most robust prediction tool for miRNA target genes. As detailed in the Methods section, we used only the validated targets with strong experimental evidence, that is, reporter assay, Western blot, or qPCR, and left out all predicted ones. In total, we identified the 43 validated target genes for miR-138-5p listed in Table 4.

**Table 4.** The Validated Target Genes of the Deregulated miRNA-138-5p With Strong Evidenced Validation

Upregulated miRNA	Strong Evidenced Validated Target Genes
miR-138-5p	*TERT*, *ROCK2*, *RHOC*, *GNAI2*, *EID1*, *FOSL1*, *BLCAP*, *MXD1*, *CCND3*, *PTK2*, *SIRT1*, *H2AFX*, *HIF1A*, *SOX4*, *EZH2*, *CASP3*, *RELN*, *S100A1*, *CDH1*, *SNAI2*, *SUZ12*, *ZEB2*, *VIM*, *RARA*, *SENP1*, *RMND5A*, *CCND1*, *ADGRA2*, *MAP3K11*, *EIF4EBP1*, *TWIST2*, *FERMT2*, *LCN2*, *CD274*, *NFKB1*, *AKT1*, *YAP1*, *BAG1*, *FOXC1*, *BCL11A*, *SOX9*, *KDM5C*, *CYTOR*

### Pathway Analysis

**Figure 4.**
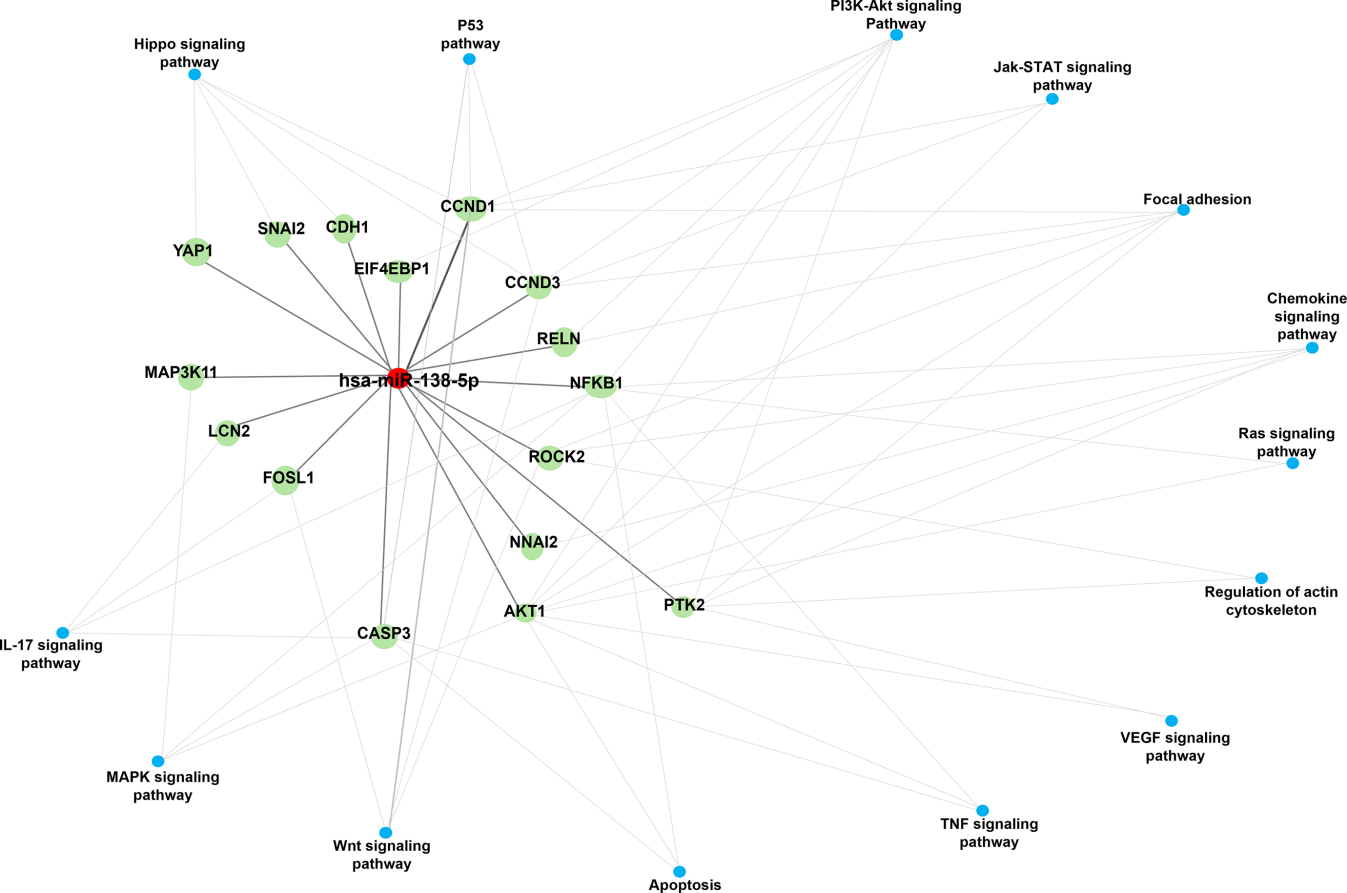
**Network map of KEGG enriched pathway of miR-138-5p.** The graphical representation of hsa-miR-138-5p target genes and 14 significant enriched KEGG pathways. The red node indicates miR-138-5p, the *green* nodes indicate miR-138-5p- target genes and the *blue* nodes interconnected to the target genes are KEGG enriched pathways.

The pathways that are significantly enriched for the target genes of miR-138-5p were identified by performing an over-representation analysis using the above-mentioned target genes as input list in GeneTrail. In total, significant enrichment for 14 KEGG pathways was found for validated gene targets of the upregulated miR-138-5p, which are displayed in Table 5. The significant 14 KEGG enriched pathway was graphically represented in a network map with their respective target genes that are predicted to be involved with the enriched pathways (Fig. 4). These pathways include PI3K-Akt signaling Hippo signaling pathway, Wnt signaling pathway, focal adhesion signaling pathway, p53 signaling pathway, IL-17, Jak-STAT, and cAMP signaling pathway. Interestingly, the target genes summarized in Table 4 – *AKT1*, *CCND1*, *CCND3*, *CASP3*, and *ROCK2* – are integral components of many of these pathways.

**Table 5.** Significantly Enriched Pathways and Associated Validated Target Genes of miR-138-5p

Name	Expected Number of Genes	Observed Number of Genes	Adjusted *P* Value	Validated Target Genes
PI3K-Akt signaling pathway	0.765804	7	5.74E-004	*AKT1*, *CCND1*, *CCND3*, *EIF4EBP1*, *NFKB1*, *PTK2*, *RELN*
Focal adhesion	0.431714	6	3.17E-004	*AKT1*, *CCND1*, *CCND3*, *PTK2*, *RELN*, *ROCK2*
Hippo signaling pathway	0.331921	5	9.96E-004	*CCND1*, *CCND3*, *CDH1*, *SNAI2*, *YAP1*
Chemokine signaling pathway	0.41002	5	1.96E-003	*AKT1*, *GNAI2*, *NFKB1*, *PTK2*, *ROCK2*
IL-17 signaling pathway	0.203925	4	1.96E-003	*CASP3*, *FOSL1*, *LCN2*, *NFKB1*
Wnt signaling pathway	0.347107	4	8.07E-003	*CCND1*, *CCND3*, *FOSL1*, *ROCK2*
MAPK signaling pathway	0.637808	4	3.84E-002	*AKT1*, *CASP3*, *MAP3K11*, *NFKB1*
p53 signaling pathway	0.156198	3	9.57E-003	*CASP3*, *CCND1*, *CCND3*
TNF signaling pathway	0.242975	3	2.39E-002	*AKT1*, *CASP3*, *NFKB1*
Apoptosis	0.292871	3	3.47E-002	*AKT1*, *CASP3*, *NFKB1*
Jak-STAT signaling pathway	0.351445	3	5.04E-002	*AKT1*, *CCND1*, *CCND3*
VEGF signaling pathway	0.127996	2	6.35E-002	*AKT1*, *PTK2*
Regulation of actin cytoskeleton	0.462086	2	4.24E-001	*PTK2*, *ROCK2*
Ras signaling pathway	0.501135	2	4.83E-001	*AKT1*, *NFKB1*

### GO Terms Enrichment Analysis Results

**Figure 5.**
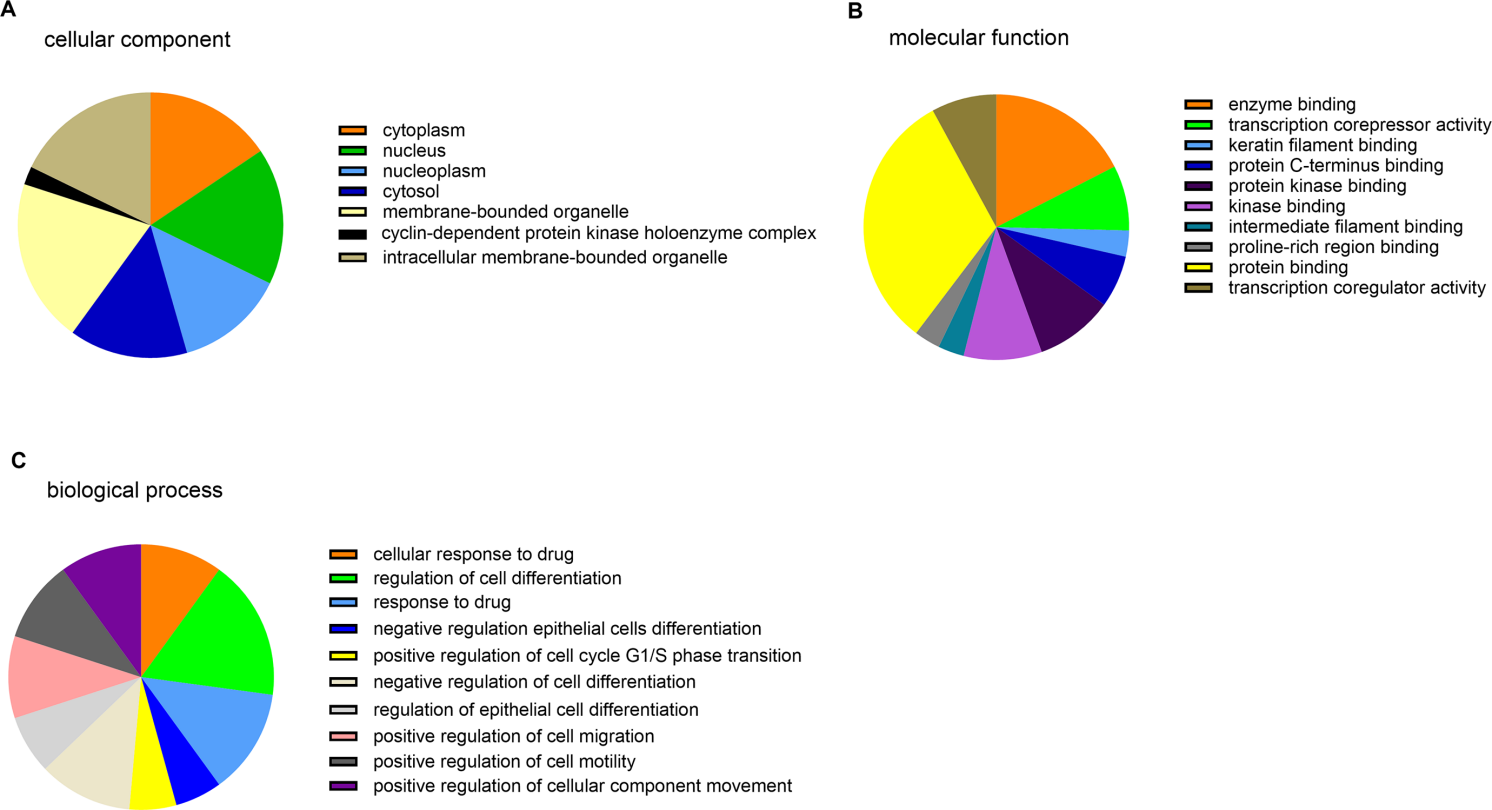
**Gene Ontology (GO) terms enrichment analysis of validated target genes.** The pie chart demonstrated GO analysis that revealed the most significant functional enrichments in cellular component (**A**), molecular function (**B**), and biological process (**C**), see also Table 6.

As detailed in the Methods section, biological significance of validated targets was explored by GO term enrichment analysis including biological process (BP), cellular component (CC), and molecular function (MF). An enrichment GO term analysis in Figure 5 shows the overview of the 10 most significant functional enrichment in CC, MF, and BP (see Figs. 5A, 5B, 5C).

We discovered that the most enriched GO for the biological process included regulation of cell differentiation, positive regulation of cell migration, and cell motility (Table 6A). For the cellular component, the genes are significantly enriched in the cytoplasm, nucleus, nucleoplasm, cytosol, membrane-bounded organelle, cyclin-dependent protein kinase holoenzyme complex, and an intracellular membrane-bounded organelle with a significant *P* value (Table 6B). About molecular function, the largest number of genes are significantly enriched in protein binding, organic cyclic compound binding, and enzyme binding (Table 6C). These significantly enriched terms and pathways could help us to further understand the role of miRNAs and their validated target genes in AAK development and progression.

**Table 6.** Gene Ontology (GO) Terms Enrichment Analysis Result of Validated Targets of miRNA 138-p

A) GO Biological Process			
Name	Expected Number of Genes	Expected Score	*P* Value
Cellular response to drug	7	0.327380	0.000040
Regulation of cell differentiation	12	1.886940	0.000040
Response to drug	9	0.817920	0.000040
Negative regulation of epithelial cell differentiation	4	0.043439	0.000097
Positive regulation of cell cycle G1/S phase transition	4	0.048736	0.000119
Negative regulation of cell differentiation	8	0.735281	0.000153
Regulation of epithelial cell differentiation	5	0.150446	0.000159
Positive regulation of cell migration	7	0.528682	0.000207
Positive regulation of cell motility	7	0.555169	0.000254
Positive regulation of cellular component movement	7	0.571061	0.000275
Negative regulation of cellular metabolic process	12	2.642350	0.000375
**B) GO Cellular Component**			
Cytoplasm	14	4.739060	0.002
Nucleus	15	5.331320	0.002
Nucleoplasm	12	3.851220	0.005
Cytosol	13	5.341910	0.01
Membrane-bounded organelle	18	10.152000	0.01
Cyclin-dependent protein kinase holoenzyme complex	2	0.042379	0.02
Intracellular membrane-bounded organelle	16	8.418650	0.02
**C) GO Molecular Function**			
Enzyme binding	11	2.301200	0.0008
Transcription corepressor activity	5	0.251097	0.0008
Keratin lament binding	2	0.006357	0.003
Protein C-terminus binding	4	0.200242	0.003
Protein kinase binding	6	0.684426	0.003
Kinase binding	6	0.771303	0.005
Intermediate lament binding	2	0.015892	0.007
Proline-rich region binding	2	0.020130	0.009
Protein binding	20	12.363100	0.009
Transcription coregulator activity	5	0.594370	0.009
bHLH transcription factor binding	2	0.030725	0.016
Transcription factor binding	5	0.700318	0.016
Protein-containing complex binding	6	1.141060	0.020
RNA polymerase II regulatory region DNA binding	5	0.821099	0.026
RNA polymerase II regulatory region sequence-specific DNA Binding	5	0.813682	0.026
Sequence-specific DNA binding	6	1.236420	0.026
Cyclin-dependent protein serine/threonine kinase regulator activity	2	0.049796	0.026
Transcription regulatory region sequence-specific DNA binding	5	0.869835	0.029887
Sequence-specific double-stranded DNA binding	5	0.919631	0.036
RNA polymerase II proximal promoter sequence-specific DNA Binding	4	0.553050	0.038
Organic cyclic compound binding	13	6.230820	0.038
Proximal promoter sequence-specific DNA binding	4	0.570002	0.038
Transcription regulatory region DNA binding	5	0.990616	0.04
Double-stranded DNA binding	5	1.014980	0.04

GO analysis revealed the largest number of genes significantly enriched in the biological process (A) cellular component (B) and molecular function (C).

### Investigation of Validated Target Genes of miRNA-138-5p Using RT-qPCR

**Figure 6.**
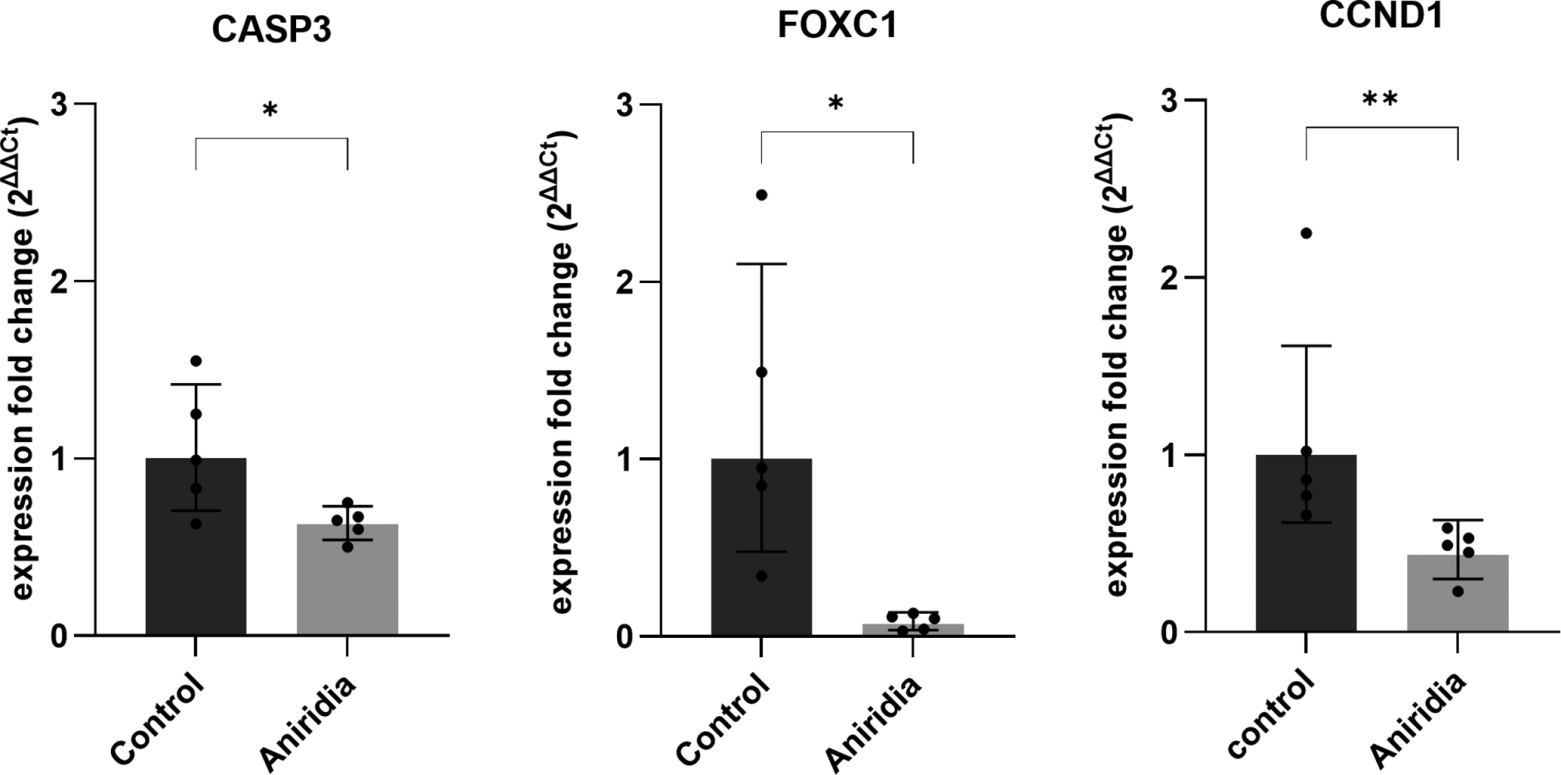
**The qPCR analysis revealed the most affected expression fold changes of the target genes of miR-138-5p in primary limbal epithelial cells (pLECs) of patients with aniridia versus healthy controls.** Data are represented as geometric mean ± geometric SD. Unpaired two-tailed *t*-test was used for normal distribution and Mann Whitney test for not normal distribution (* *P* < 0.05, ** *P* < 0.01). The qPCR results revealed that 3 direct target genes of miR-138-5p, caspase 3 (*CASP3*) (*P* < 0.05), Forkhead Box C1 (*FOXC1*) (*P* < 0.05) and Cyclin D1 (*CCND1*) (*P* < 0.01) were significantly reduced in aniridia samples compared with controls.

We examined the validated target genes of miRNA-138-5p, specifically *AKT1*, *CASP3*, *FOSL1*, *CCND1*, *CCND3*, *ROCK2*, *YAP1*, *MAPK3*, and *FOXC1*, out of 43 identified target genes listed in Table 4 in the 5 included additional congenital aniridia samples. These genes were selected based on their potential association with AAK pathogenesis or insights from pathway analysis. The qPCR results revealed that 3 direct target genes of miR-138-5p, caspase 3 (*CASP3*; *P* < 0.05), Forkhead Box C1 (*FOXC1*; *P* < 0.05), and Cyclin D1 (*CCND1*; *P* < 0.01) were significantly reduced in aniridia samples compared with controls (Fig. 6). The remaining target genes did not exhibit significant differences between the sample groups.

## Discussion

To the best of our knowledge, this is the first study that has been able to provide a starting point for miRNA research with respect to pLECs of patients with aniridia compared with healthy controls. This study attempted to develop valuable clues to understand miRNA profiles and identification of expression changes at the miRNA level in pLECs. Furthermore, we focused not only on miRNAs but also on the reliable set of validated targets from miRTargetLink and miRTarbase with strong experimental evidence to find changes in pathways, which may be associated with AAK.

Our previous study by Latta et al. found evidence for an overall altered miRNA expression pattern in conjunctival cells of patients with aniridia, compared with healthy controls using miRNA microarray.^24^ In that study, 21 significantly deregulated miRNAs were identified in conjunctival cells of 20 subjects with congenital aniridia, relative to controls (fold change ≤ –1.5 or ≥ +1.5, adjusted *P* < 0.05). This study reported that differential expression of miRNAs in conjunctival cells of patients with aniridia was associated with highly neovascularized corneas observed in severe AAK.^24^ Several further studies reported the major role of miRNAs in corneal angiogenesis,^36^ corneal wound healing,^37^ corneal neovascularization,^19^^,^^20^^,^^23^ and corneal differentiation.^38^^,^^39^ Nevertheless, the differentially expressed miRNAs of the previous study analyzing conjunctival cells of patients with aniridia did not overlap with deregulated miRNAs in limbal epithelial cells of patients with congenital aniridia of the present study.

In the present study, miRNA profiles have been obtained from limbal biopsies of five patients with aniridia and five healthy subjects using miRNA microarray. In large-scale multiple testing (often by microarray analysis), the controlling of the FDR is required, which is defined as the expected value of the false positives among all significant results.^40^^,^^41^ To control the FDR and identify which *P* values remain significant after correction, adjusted *P* values were calculated following the Benjamini Hochberg correction method. The results showed that no miRNAs remain significantly dysregulated following correction. However, the low number of samples is the limiting factor of this study that might have contributed to not significant *P* values within the data set. Few published studies addressed the significantly altered expression of corneal miRNAs obtained from larger sample size in each group indicating the impact of sample size on statistical tests. A study by Teng et al., identified 18 differentially expressed miRNAs, among them, 14 were in the human limbal epithelium, compared to the central corneal epithelium of 9 human corneas.^42^ The study by Latta et al., identified 21 significantly deregulated miRNAs in conjunctival cells of 20 subjects with aniridia relative to controls.^24^

Although, in this present study, there was no significant difference in the expression of miRNAs in pLECs after correction of the *P* value, the data generated serves as a platform to study the miRNAs, which might play a role in the pathogenesis of AAK. Nevertheless, we observed that pLECs in aniridia exhibit altered miRNA expression, however, only before the Benjamini Hochberg correction of the *P* value. The results demonstrated 10 differentially expressed miRNAs under the rule of a |log2 (fold change)| > +2, < –2 and a raw *P* < 0.05. Among them, miR-138-5p (fold change = 6.67) was strongly upregulated, and miR-409-3p (fold change = –8.83), miR-495-3p (fold change = –8.42), and miR-127-3p (fold change = –8.09) were strongly downregulated compared with healthy controls. For microarray validation and more confidence in the accuracy of the microarray results, qPCR was used. Validation of the microarray data using RT-qPCR with the same sample set revealed a significantly higher expression of miR-138-5p (fold change = 2.55). Thereafter, a similarly significant elevation in miR-138-5p expression (fold change = 5.33) was observed when an additional sample set comparing patients with aniridia with controls was included, further validating the reliability of the microarray results. The patients with aniridia included in the study exhibited different AAK grades; nevertheless, miR-138-5p expression did not differ significantly between the subgroups. However, significant discrepancies were noted between microarray and qPCR results. Although qPCR is commonly used for validating microarray analyses, miRNA fold change data often shows notable differences between the two methods. A literature review by Morey et al.^43^ indicated that the correlation between microarray and qPCR data can range widely, from –0.48 to 0.94. Various studies have investigated factors contributing to these differences. For instance, Wurmbach et al.^44^ and Rajeevan et al.^45^ found that genes exhibiting less than a two-fold change often show lower correlation between microarray and qPCR analyses. Additionally, factors such as variations in primer location between microarray and qPCR can impact the correlation between these methods.^46^ Nevertheless, the qPCR analysis indicated the same direction of expression change for most of the miRNAs as indicated in microarray. The microarray and qPCR results of the present study showed that there is no overlapping expression between the differentially expressed miRNAs in pLECs and in conjunctival cells that are reported in our previous study,^24^ likely due to the differences in sample preparation. The conjunctival sample obtained using EYEPRIM tool in the previous study comprised different cell types, including pLECs, whereas, in the current study, cultured pLECs were used for miRNA profiling.

Kalaimani et al. demonstrated 38 miRNAs highly expressed in human corneal epithelial stem cells (CESCs) and 301 miRNAs highly expressed in differentiated central corneal epithelial cells (CCECs).^47^ However, as there is no overlapping expression found between deregulated miRNAs in CESCs and CCECs, it can be hypothesized that this dissimilarity could correlate with differentiation status of the cells. The single cell RNA sequencing (scRNA-seq) studies have reported a comprehensive analysis of the human cornea, and limbal and conjunctival cells. Providing the differentiation status of these cell types,^48^^,^^49^ miR-31 was found higher in CCECs than LECs, which was reported as differentiation-specific miRNA.^20^ Another example is miR-184, which has a unique role in regulation of differentiation of corneal epithelial cells.^39^ Furthermore, miR-184 is expressed in the basal and suprabasal layers of the corneal epithelium but not in the limbal epithelium.^50^^–^^52^ Interestingly, the number of expressed miRNAs in differentiated cells, for example, CCECs is higher than the number of miRNAs expressed in undifferentiated cells, for example, CESCs.^47^^,^^49^ Therefore, comparing the differential expression of 21 miRNAs in differentiated conjunctival cells and 301 miRNAs in differentiated central corneal epithelial cells from previous published studies with only differential expression of 10 miRNAs in mostly undifferentiated pLECs (unadjusted *P* value) in this present study, appear that miRNAs expression is largely dependent on the differentiation status of the cells. As described in the literature, miRNAs regulate many aspects of development, differentiation, angiogenesis, and corneal neovascularization in different cell types.^18^^,^^23^^,^^31^^,^^34^^,^^38^

One particularly interesting miRNA observed in this study is the miR-138-5p, which was upregulated in microarray and this upregulation could also be validated by RT-qPCR. The miR-138-5p is a critical miRNA in tumorigenesis and has been demonstrated to serve as a tumor suppressor in retinoblastoma (RB).^53^^,^^54^ Functionally, the overexpression of miR-138-5p represses cell viability, migration, and invasion, and induces apoptosis of RB cells through suppressing pyruvate dehydrogenase kinase 1 (PDK1).^55^ It has been reported that miR-138-5p can inhibit the malignant progression of prostate cancer^56^ and lung cancer growth through the miR-138-5p/FOXC1 pathway.^57^ Yuan et al. demonstrated that miR-138-5p negatively regulates FOXC1, promoting IL-1β-induced cartilage degradation.^58^ However, to our knowledge, there are no reports on the role of the miR-138-5p/FOXC1 pathway in the pathogenesis of AAK. RT-qPCR analysis of miR-138-5p target genes revealed a marked reduction in FOXC1 levels in aniridia samples. These findings suggest that the reduced FOXC1 gene expression in aniridia may be a response to the upregulation of miR-138-5p. Interestingly, Seo et al. reported that FOXC1 expression regulates VEGF signaling, which is crucial for maintaining corneal avascularity.^59^^,^^60^ FOXC1 has been identified as a direct downstream target of PAX6 through differential gene expression analysis in the developing eyes of wildtype and PAX6^±^ mice.^61^ FOXC1 is co-expressed with PAX6 in the limbus and central cornea, and PAX6 expression was shown to be inhibited in FOXC1 knockdown LSCs.^62^ Furthermore, FOXC1 overexpression in PAX6^±^ mice enhanced limbal stem cell proliferation after corneal injury.^63^ These findings suggest that dysregulation of the FOXC1 gene could contribute to abnormal angiogenesis and inhibited cell proliferation, supporting the hypothesis that reduced FOXC1 expression in aniridia may be a response to the upregulation of miR-138-5p. This highlights the role of the FOXC1/miRNA-138-5p pathway in the pathogenesis of AAK.

In addition to FOXC1, caspase 3 levels were reduced in aniridia pLECs compared with healthy controls. In human multiple myeloma cells, miR-138-5p negatively targets caspase 3 to regulate apoptosis and proliferation of the cells.^64^ In hippocampal neurons, overexpression of miR-138-5p reduces caspase dependent apoptosis, thereby favoring the survival of the cells.^65^ Thus, in aniridia samples, the reduction in caspase 3 mRNA expression could also be regulated by the overexpression of miR-138-5p. Nevertheless, not much is known for the role of caspases in aniridia or limbal stem cell proliferation. Recent studies have reported diverse functions of caspases that are not limited to apoptosis. Fujita et al. and Janzen et al., suggested that caspase 3 might be involved in stem cell differentiation.^66^^–^^68^ Pathway enrichment analysis revealed that caspase 3 is associated with p53, IL-17, MAPK signaling, and apoptosis. However, further functional studies are required to uncover the contribution of miR-138-5p dependent Caspase 3 activity in apoptosis or cell proliferation in pLECs.

Pathway analysis also highlighted the main involvement of the Hippo, Wnt, Focal adhesion, cAMP, and p53 signaling pathways. The validated target genes *CCND1*, *CCND3*, and *YAP1* (regulated by miR-138-5p) were associated with the Hippo signaling pathway. Particularly in regulating Wnt, Focal adhesion, and p53 signaling pathways, miR-138-5p dysregulated the pathways through the target genes *CCND1*, *CCND3*, and *ROCK2*, and the cAMP signaling pathway by *GNAI2*, *ROCK2*, and *SOX9*. There is already evidence of alterations in Hippo, Wnt, and Focal adhesion signaling pathways in AAK.^18^^,^^69^^,^^70^ It has been shown that ablation of PAX6 leads to an increased expression of the cell cycle promoting gene CyclinD1 (*CCND1*) in retinal progenitor cells (RPCs), indicating a central role of PAX6 in regulation of cell cycle exit during differentiation.^71^ The cell cycle regulator CCND1 plays a central role in the stem cell expansion of various progenitor cells and inhibits differentiation.^72^ Quiescent cells enter the cell cycle upon CCND1 stimulation in response to mitogenic signals from the LESC niche.^73^^,^^74^ Additionally, CCND1 is a well-established transcriptional target of the Hippo-Yap signaling pathway, which regulates limbal stem cell proliferation.^75^^,^^76^ In aniridia pLECs compared with healthy controls, the expression of CCND1 was significantly reduced. Given that PAX6 is typically depleted or mutated in AAK, the upregulation of miR-138-5p may represent a compensatory mechanism by the cells to lower protein levels of its target, CCND1, and restore control over cell cycle progression. Alternatively, the upregulation of miR-138-5p could suppress the mitogenic signals from the LESC microenvironment, reducing CCND1 levels and inhibiting limbal stem cell proliferation. Thus, further research is needed to elucidate the PAX6/miR-138-5p/CCND1 axis. Taken together with previous findings, our target gene analysis in this study suggests that highly expressed miRNAs in pLECs, including those in stem cells, may play a role in regulating limbal stem cell function. Additionally, limited information is currently available on miRNAs that are constitutively and specifically expressed in AAK. Therefore, further studies are required to explore the functional role of these identified miRNAs and their targets in the pathogenesis of AAK.

Limitations of the present study are the low sample size, and the age difference of patients with congenital aniridia and healthy limbal sample donors, due to the fact that aniridia is a rare disease. Therefore, collecting a large number of samples requires a waiting period of several years. Nevertheless, patients may not give informed consent for research purposes for personal reasons. In addition, after collection, the isolation and culturing of the desired cells could also be affected by the sample size of the study. The present study provides a research basis on identified miRNAs in pLECs, nevertheless, future studies on in vitro and in vivo cell models are required to investigate the possible mechanism of these miRNAs.

## Conclusions

In this study, we demonstrate that both healthy and aniridia primary limbal epithelial cells express low levels of miR-204-5p. Through our microarray profiling, we identified a novel microRNA (miR-138-5p) that is upregulated in aniridia samples, with reduced expression of its validated target genes, *FOXC1*, *CASP3*, and *CCND1*. PI3K-Akt, Hippo, Wnt, Focal adhesion, cAMP, p53, IL-17, Jak-STAT, and MAPK-signaling pathways emerged as the most enriched pathways associated with miR-138-5p. These findings may be significant in uncovering the pathogenesis of AAK. However, further research is needed to clarify the direct relationship between miR-138-5p and limbal stem cell functions.

## Supplementary Material

Supplement 1
