# Supplementary material for: miRNA Expression Profile in Primary Limbal Epithelial Cells of Aniridia Patients
Source: Invest Ophthalmol Vis Sci. 2025 Jan 9;66(1):20. doi: 10.1167/iovs.66.1.20 (PMC11725988; doi:10.1167/iovs.66.1.20)
Supplement: Supplement 1 [file iovs-66-1-20_s001.pdf]

Table S1. Qiagen miRCURY LNA miRNA primer pairs used for qRT-PCR.

miRNA transcript	Order number (Qiagen)	
miR 495-3p	5'AAACAAACAUGGUGCACUUCUU	(YP00206015)
miR 409-3p	5'GAAUGUUGCUCGGUGAACCCCU	(YP00204358)
miR 127-3p	5'UCGGAUCCGUCUGAGCUUGGCU	(YP00204048)
miR 493-5p	5'UUGUACAUGGUAGGCUUUCAUU	(YP00204166)
miR 138-5p	5'AGCUGGUGUUGUGAAUCAGGCCG	(YP00206078)
miR 103a-3p	5'AGCAGCAUUGUACAGGGCUAUGA	(YP00204063)
UniSp6		339390
U6 snRNA		YP02119464

Table S2. Primer pairs used for qPCR.

AKT1 138bp (NM_001014431),138 (NM_001014432),138 (NM_005163),138 (XM_005267401)	QT00085379
CASP3 147bp (NM_004346),147 (NM_032991)	QT00023947
FOSL1 150bp (NM_005438)	QT00043596
CCND1 96bp (NM_053056),96 (XM_006718653)	QT00495285
CCND3 138bp (NM_001760)	QT00096796
ROCK2 82bp (NM_004850),82 (XM_005246190)	QT00011165
YAP1 109bp (NM_001130145),109 (NM_001195044),109 (NM_001195045),109 (NM_006106),109 (NM_001282097),109 (NM_001282098),109 (NM_001282099),109 (NM_001282100),109 (NM_001282101),109 (XM_005271378),109 (XM_005271380),109 (XM_005271381),109 (XM_005271383)	QT00080822
MAP3K11 69bp (NM_002419)	QT00084749
FOXC1 109bp (NM_001453)	QT00217161
